# Aflatoxins in food products consumed in the Kingdom of Saudi Arabia: A preliminary dietary risk assessment

**DOI:** 10.1002/fsn3.3526

**Published:** 2023-06-27

**Authors:** Jumanah Alamir, Lama Almaiman, Yasser Alrujib, Rayan Alhamidi, Bandar Alowais, Saqer Alhussain, Abdullah Aldakheelallah, Majid Alkhalaf, Mohammed Bineid

**Affiliations:** ^1^ Department of Monitoring and Risk Assessment, Food Sector Saudi Food and Drug Authority Riyadh Saudi Arabia; ^2^ Executive Department of Laboratories, Research and Laboratories Sector Saudi Food and Drug Authority Riyadh Saudi Arabia; ^3^ National Nutrition Committee Saudi Food and Drug Authority Riyadh Saudi Arabia

**Keywords:** aflatoxins, cancer risk, contamination, risk assessment

## Abstract

Aflatoxins (AFs) are hepatotoxic, mutagenic, genotoxic, and immunosuppressive toxins. Several food commodities consumed in the Kingdom of Saudi Arabia (KSA) are susceptible to AF contamination because of improper storage practices and the warm and humid climate of the country. Therefore, the occurrence of AFs in 2388 food samples was measured and the estimated daily intake (EDI) of AFs in Saudi adults was assessed. The risks of AFB_1_ exposure were characterized using the margin of exposure (MoE) approach and by estimating the number of possible hepatocellular carcinoma (HCC) cases in the KSA. The results revealed that 12.1% of the analyzed samples were contaminated with AFs and the highest concentration of total AFs was observed in the nut and seed group. The mean EDI of AFB_1_ was estimated to be 0.21 and 0.55 ng/kg body weight (bw)/day for the lower bound (LB) and upper bound (UB) scenarios, respectively. The MoEs were estimated to be 1902.4 and 722.1, while the estimated liver cancer risk ranged from 0.002 to 0.008 cancer cases/year/100,000 persons. Based on the study's findings, contamination with AFs in the KSA is low; however, AFs are considered potent genotoxic contaminants, and therefore, exposure through food should be kept as low as possible.

## Graphical abstract

Dietary risk assessment of aflatoxins for the adult population in the Kingdom of Saudi Arabia.

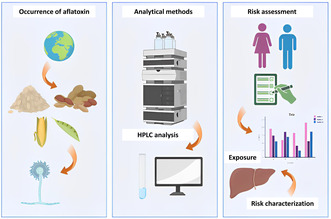

## 1 INTRODUCTION

Aflatoxins (AFs) are highly toxic secondary metabolites produced by certain fungal species of *Aspergillus*, namely, *Aspergillus flavus* and *Aspergillus parasiticus* (Ellis et al., 1991; Udovicki et al., 2018). Food contamination with AFs is a worldwide concern owing to the huge economic losses and their impact on human health (Eskola et al., 2020). Therefore, most food safety regulatory agencies apply strict regulations to minimize human dietary exposure. Several types of AFs have been identified, but four of them naturally occur, namely, aflatoxin B_1_ (AFB_1_, AFB_2_, AFG_1_, and AFG_2_), which is known to be hazardous to human health (Kos et al., 2013). Commodities contaminated with AFs include grains, spices, cereal crops such as rice and wheat, and tree nuts such as peanuts and pistachios (Inan et al., 2007). Food contamination with AFs is mainly due to fungal infections in both the pre‐ and postharvest stages. Climatic conditions, such as humidity, rainfall, and high temperatures, help facilitate fungal growth and production of AFs (Cotty & Jaime‐Garcia, 2007; Mahato et al., 2019). Poor storage and transportation conditions also contribute to the occurrence of AFs in food (Abdullah AlFaris et al., 2020). Normal home‐cooking processes cannot destroy AFs because of their heat‐resistant nature (Abrehame et al., 2023; Kumar et al., 2016).

Ingestion of AFs is associated with serious life‐threatening health conditions (Kumar et al., 2016; Mahato et al., 2019). Epidemiological studies have shown that long‐term exposure to AFs can lead to immunosuppression, liver cirrhosis, growth impairment, and teratogenicity (Ismail et al., 2021; Kuniholm et al., 2008). The International Agency for Research on Cancer (IARC) has classified AFs as carcinogens, with AFB_1_ being the most potent carcinogen and classified as a Group 1 carcinogen in humans, causing hepatocellular carcinoma (HCC; Theumer et al., 2018). In the Kingdom of Saudi Arabia (KSA), liver cancer ranks fifth among cancers in males and ninth in females (Alghamdi & Alghamdi, 2020). The IARC estimated the age‐specific incidence rate (ASIR) of liver cancer in the KSA to be 5.2 per 100,000 people (Bray et al., 2018). Owing to these health concerns, many food regulatory agencies have set maximum limits (MLs) for AFs in food. The Codex Alimentarius Commission has set an ML of 15 μg/kg for total AFs in unprocessed nuts and 10 μg/kg for ready‐to‐eat nuts (Codex Alimentarius FAO‐WHO). In the European Union (EU), total AFs are regulated at several levels ranging from 4 μg/kg to 15 μg/kg depending on the foodstuffs (The European Union. Commission Regulation (EC), 2006). In the USA, the maximum limit for AFs in all foods is 20 μg/kg (U.S. Food and Drug Administration). In the KSA, the total AFs should not exceed 10 μg/kg in ready‐to‐eat nuts and spices. In grains, dried fruits, and their products, the ML is set at 4 μg/kg, and it is 20 μg/kg in other food commodities that are not listed in the standard (GCC Standardization Organization).

Varying levels of AFs have been reported for many food products consumed in the KSA. Commodities of the most commonly consumed food items such as cereals and grain products, nuts and seeds, legumes, coffee, baked goods, and spices have been reported to contain AFs (Abdullah AlFaris et al., 2020; Bokhari, 2001; El Tawila et al., 2020; Serdar et al., 2020). The Saudi Food and Drug Authority (SFDA) has established an annual National Food Monitoring Program (NFMP) to measure contaminant levels in food and check compliance with established MLs. AFB_1_, AFB_2_, AFG_1_, and AFG_2_ were the target contaminants in this program. More than 2000 samples were analyzed for their AF levels within the annual monitoring plan set by the SFDA; however, these results have never been used to estimate dietary exposure and assess the risks related to exposure to these levels.

In this study, the results obtained from the NFMP for the period of 2015–2020 were used. The occurrence levels of AFs in four food groups (cereals and grains; nuts and seeds; pulses, legumes, beans, and their products; and spices) were extrapolated. Consequently, the estimated daily intake (EDI) of AFs for the Saudi adult population was assessed, and the potential health risks were evaluated using margin of exposure (MoE) and cancer risk approaches.

## 2 MATERIALS AND METHODS

### 2.1 Sampling

The analytical data of 2388 food samples from the NFMP from 2015 to 2020 were used in this study. The samples analyzed under the NFMP were collected from different regions in the KSA according to the Standardized Sampling and Transporting Procedures, which are in compliance with the Codex Alimentarius Commission general guidelines on sampling (Codex Alimentarius FAO‐WHO), taking into consideration laboratory capabilities. Most (80%) of the food items were collected from the central, western, and eastern regions of the KSA, as these regions represent around 80% of the population distribution in the administrative regions of the KSA. Samples were collected from food factories, storage facilities, and distribution centers (1 kg at least of each sample). The samples were placed in clean and suitable packaging and sent to the SFDA laboratories (Riyadh Laboratory, Jeddah Laboratory, or Dammam Laboratory). Both domestic and imported samples were included in the analysis. Domestic samples are raw commodities that are grown in other countries but are processed, prepared, packed locally by domestic food manufacturers, and sold under Saudi brand names. The imported samples were food commodities that entered the KSA as final products. The sample characteristics are listed in Table 1.

**TABLE 1:** Number of domestic and imported samples analyzed for aflatoxins during the period of 2015–2020.

Food group	Food items	Total number of samples	No. of domestic samples	No. of imported samples	No. of unknown‐origin samples
Cereals and grains	Breakfast cereals	50	0	50	0
	Cake	25	19	5	1
	Corn	20	7	4	9
	Dessert mixes	46	23	23	0
	Groats	17	5	12	0
	Oats	9	0	6	3
	Pasta	53	20	32	1
	Popcorn	25	11	14	0
	Rice	164	33	120	11
	Semolina	38	9	28	1
	Wheat	22	7	12	3
	Wheat flour	75	44	31	0
Nuts and seeds	Almonds	160	35	125	0
	Cashews	145	43	79	23
	Halawa tahiniah	6	1	5	0
	Hazelnuts	30	13	14	3
	Pistachios	432	94	284	54
	Sunflower seeds	46	20	24	2
	Walnuts	62	24	38	0
	Peanut butter	91	7	66	18
	Other nuts and seeds	169	80	85	4
Pulses, legumes, beans, and their products	Beans	63	16	45	2
	Broad beans	19	11	6	2
	Chickpeas	128	24	93	11
	Cowpeas	44	15	26	3
	Lentils	85	18	54	13
	Peanuts	214	102	92	20
	Peas	40	9	29	2
	Soybeans	31	3	26	2
Spices	Spices	79	46	31	2
Total number of samples analyzed		2388	739	1459	190

### 2.2 AFs analytical method

All NFMP samples were analyzed in SFDA laboratories accredited under ISO/IEC 17025 (International Organization for Standardization (ISO). ISO/IEC 17025, 2017). AFB_1_, AFB_2_, AFG_1_, and AFG_2_ were quantified using high‐performance liquid chromatography (HPLC) with immunoaffinity column cleanup and postcolumn derivatization, as described in ISO 16050:2003 (International Organization for Standardization (ISO)). First, 25 g of each test sample was homogenized with 5 g of sodium chloride (NaCl) (NEN Tech LTD), and 125 mL of water/methanol (H_2_O/MeOH) (LiChrosolv) at a ratio of 30/70 (v/v) was added to the homogeneous test sample. The extraction protocol involved the use of an immunoaffinity column specific for AFB_1_, AFB_2_, AFG_1_, and AFG_2_. The supernatant was eluted with methanol before injection into the instrument.

The operation conditions of the instrument involve separation using a C18 column (Kinetex 2.6 μ; C18, 100 mm × 3μ) (Phenomenex), with a flow rate of 0.3 mL/min and temperature at 60°C. The instrument was equipped with a fluorescence detector at wavelengths of 362 nm and 455 nm for excitation and emission, respectively, and was equipped with postcolumn derivatization with pyridinium hydrobromide perbromide (PBPB, 0.05 g/1 L) (Sigma‐Aldrich) for the determination of AFB_1_ and AFG_1_.

### 2.3 Quality assurance

The laboratory used a combination of certified reference materials (CRM), reference materials (RM), quality control samples, and blank samples as tools to ensure the validity of the results. In addition, quality control programs are implemented to ensure the validity of test results, such as participation in proficiency test schemes (PT), calibration programs, preventive maintenance programs, and the use of statistical analysis. The validation parameters included the limit of detection (LOD), limit of quantification (LOQ), linearity, and mean recovery (Table 2).

**TABLE 2:** Summary of the validation parameters used for aflatoxin analytical methods.

Food group	Analyte	LOD (μg/kg)	LOQ (μg/kg)	Linearity (*R* ^2^)^a^	Mean recovery (%)
Cereals and grains	AFB_1_	0.09	0.26	0.999	78.13
	AFB_2_	0.02	0.05	0.999	98.29
	AFG_1_	0.20	0.61	0.999	74.67
	AFG_2_	0.06	0.17	0.999	91.88
Nuts and seeds	AFB_1_	0.28	0.84	0.999	106.00
	AFB_2_	0.05	0.16	0.999	102.40
	AFG_1_	0.19	0.56	0.999	106.40
	AFG_2_	0.06	0.17	0.999	99.20
Pulses, legumes, beans, and their products	AFB_1_	0.09	0.26	0.999	93.53
	AFB_2_	0.02	0.05	0.999	103.73
	AFG_1_	0.20	0.61	0.999	93.73
	AFG_2_	0.06	0.17	0.999	96.27
Spices	AFB_1_	0.26	0.86	0.999	93.29
	AFB_2_	0.05	0.18	0.999	101
	AFG_1_	0.45	1.51	0.999	104.9
	AFG_2_	0.19	0.62	0.999	102.4

^a^ *R* ^2^, coefficient of determination.

### 2.4 Estimation of daily food consumption

Owing to the lack of recent food consumption data in the KSA for use in the assessment of chronic dietary exposure to AFs, the GEMS/Food cluster diets tool developed by The World Health Organization (WHO) was used as the main source to estimate daily food consumption (The World Health Organization). Although these data have many limitations and uncertainties, they are the best available source of food consumption data for estimating dietary exposure to AFs in the KSA. A default value of 0.1 g per day was assigned in the absence of food items in the GEMS/Food cluster diets, as in the case of a food item named halawa tahiniah in the current study.

### 2.5 Exposure assessment

Only chronic EDI associated with AFs was assessed in this study. The target age group was adults aged 18–85 years old, and the average body weight was 70 kg, based on values reported in the literature (Al‐Malki et al., 2003). This study assessed chronic dietary exposure to individual AFB_1_, AFB_2_, AFG_1_, AFG_2_, and total AFs by summing the analytical results of the four AFs for each food item. For exposure assessment, the EDI and concentration units were converted to ng. Equation (1) was used to calculate the EDI.

(1) $$\mathrm{EDI}\;\left(\mathrm{ng}/\mathrm{kg}\;\mathrm{bw}/\mathrm{day}\right)=\left(\text{average concentrations}\;\left[\mathrm{ng}\right]\times \text{daily food consumption}\;\left[g/\mathrm{day}\right]\right)/1000\left(g\right)/\text{body weight}\;\left(\mathrm{kg}\right)$$

To achieve a more accurate EDI of AFs and reduce uncertainties, food items with a low number of samples (<5 samples) were grouped with similar food items to better represent the contribution of the food group to the total dietary exposure (e.g., pancake mixes were grouped with desert mixes). Additionally, the outlier results and food items represented by either a low number of samples or for which all data were below the LOD were considered unsuitable for use in the exposure assessment.

To treat left‐censored data, the substitution method was applied (European Food Safety Authority, 2010). The lower bound (LB) was obtained by substituting the numerical values of the LOQ with values reported as <LOQ and nondetected samples were assumed to have a value of zero. The upper bound (UB) was obtained by assigning numerical values of the LOQ to all values reported as not detected (ND), <LOD, or <LOQ. The substitution method was only applied to AFB_1_, AFB_2_, AFG_1_, and AFG_2_. The sum of all analytical results for the four AFs was calculated to represent the total AFs.

### 2.6 Risk characterization

Scientific evidence has proven that AFs are genotoxic and carcinogenic; therefore, no health‐based guidance values were identified. Instead, the MoE approach was applied as advised by The European Food Safety Authority (EFSA) Panel on Contaminants in the Food Chain (EFSA Panel on Contaminants in the Food Chain (CONTAM) et al., 2020). The panel selected a benchmark dose lower confidence limit (BMDL_10_) of 0.4 μg/kg body weight (bw)/day for the incidence of HCC in male rats following AFB_1_ exposure. According to the EFSA Scientific Committee, if BMDL_10_ is selected from an animal carcinogenicity study, an MoE of 10,000 or higher is considered to be of low risk to health. MoE is a ratio calculated by dividing BMDL_10_ by the exposure estimates (European Food Safety Authority (EFSA), 2005) as illustrated in Equation (2).

(2) MoE=BMDL/EDI

The cancer risk estimates following exposure to AFB_1_ were calculated considering the carcinogenic potency (*P* estimate) of AFs, following the procedures of the Food and Agriculture Organization (FAO)/WHO (Joint FAO/WHO Expert Committee on Food Additives 1997: Rome, I; Organization, W. H; Nations, F. and A. O. of the U. World Health Organization, 1999). As stated by the committee, the carcinogenic potency of AFs for individuals carrying hepatitis B virus (PHBsAg^+^) is set at 0.3 cancers/year/per 100,000 individuals, while the carcinogenic potency for noninfected individuals is set at (PHBsAg^−^) = 0.01 cancers/year/per 100,000 individuals. In this assessment, the prevalence rate of hepatitis B virus (HBV) infection (HBsAg^+^) was obtained from Aljumah et al. (Aljumah et al., 2019), where it is currently estimated to be 1.3% of the general population in the KSA. Therefore, the aflatoxin‐related HCC risk was calculated using Equation (3):

(3) $$\begin{array}{l} \;P\;\text{estimate}=\left({\text{PHBsAg}}^{+}\;\times \;\%\;\text{population}\;{\text{HBsAg}}^{+}\right)\;+\;\left({\text{PHBsAg}}^{-}\;\times \;\%\text{population}\right)\\ \text{Cancer risk estimates}=P\;\text{estimate}\;\times \;\mathrm{EDI} \end{array}$$

Where PHBsAg^+^ and PHBsAg^−^ are carcinogenic potency of AFs for hepatitis B surface antigen‐positive (HBsAg^+^) and hepatitis B surface antigen‐negative (HBsAg^−^) individuals, respectively.

### 2.7 Statistical analysis

Statistical analyses were performed using Microsoft Excel 2016. The means and standard deviations (SD) of the LB and UB were determined. A modified Z‐score box plot was constructed for the samples to identify outliers.

## 3 RESULTS

### 3.1 Occurrence of AFs

A summary of the concentrations classified by food group is shown in Table 3. The percentage of detected AFs (positive samples) was 12.1% (289 of 2388 samples).

**TABLE 3:** The occurrence of aflatoxins in four food groups extrapolated from the National Food Monitoring Program.

Food group (No. of samples)	No. of positive samples (%)	Parameter	AFB_1_	AFB_2_	AFG_1_	AFG_2_	Total AFs
Cereals and grains (*n* = 544)	50 (9.1)	Mean value LB (μg/kg) ± SD Mean value UB (μg/kg) ± SD Range (min–max, μg/kg) Mean of positive samples (μg/kg)	0.056 ± 0.272 0.295 ± 0.228 ND – 4 0.693	0.006 ± 0.111 0.075 ± 0.115 ND – 2.56 0.307	0.003 ± 0.067 0.605 ± 0.044 ND – 1.56 1.53	0.003 ± 0.064 0.172 ± 0.057 ND – 1.49 1.49	0.068 ± 0.425 1.147 ± 0.362 ND – 7.77 0.840
Nuts and seeds (*n* = 1141)	163 (14.3)	Mean value LB (μg/kg) ± SD Mean value UB (μg/kg) ± SD Range (min–max, μg/kg) Mean of positive samples (μg/kg)	0.444 ± 1.766 1.184 ± 1.592 ND – 19.5 0.834	0.070 ± 0.299 0.211 ± 0.269 ND – 4.24 0.581	0.057 ± 0.470 0.590 ± 0.415 ND – 12.08 1.802	0.041 ± 0.288 0.203 ± 0.267 ND – 4.39 0.977	0.612 ± 2.357 2.187 ± 2.094 ND – 34 3.859
Pulses, legumes, beans, and their products (*n* = 624)	64 (10.2)	Mean value LB (μg/kg) ± SD Mean value UB (μg/kg) ± SD Range (min–max, μg/kg) Mean of positive samples (μg/kg)	0.114 ± 0.494 0.510 ± 0.481 ND – 6.36 0.133	0.030 ± 0.193 0.104 ± 0.189 ND – 3.69 0.332	0.026 ± 0.202 0.607 ± 0.134 ND – 2.49 1.258	0.008 ± 0.105 0.175 ± 0.094 ND – 2.44 0.502	0.178 ± 0.888 1.396 ± 0.793 ND – 14.980 1.742
Spices (*n* = 79)	12 (15.1)	Mean value LB (μg/kg) ± SD Mean value UB (μg/kg) ± SD Range (min–max, μg/kg) Mean of positive samples (μg/kg)	0.085 ± 0.409 0.890 ± 0.270 ND – 3.26 1.34	0.025 ± 0.200 0.200 ± 0.179 ND – 1.77 0.977	0.038 ± 0.239 1.510 ± 0.00 ND – 1.51 1.51	1.025 ± 6.168 1.597 ± 6.074 ND – 43.67 13.49	1.172 ± 6.215 4.198 ± 6.086 ND – 43.67 10.3
Total no. of samples (*n* = 2388)	289 (12.1)						

*Note*: All values represent the mean ± SD for aflatoxins in the food groups.

Abbreviation: LB, lower bound; ND, not detected; SD, standard deviation; UB, upper bound.

Table S1 illustrates the mean concentrations of all four types of AFs and total AFs in the selected 30 food items. With reference to these food items, the LB mean concentrations of AFB_1_ and AFB_2_ ranged between ND and 3.8 μg/kg, and ND and 0.62 μg /kg, respectively, whereas the UB mean concentration of AFB_1_ and AFB_2_ ranged from 0.26 to 3.9 and from 0.05 to 0.64 μg/kg, respectively. In the case of AFG_1_ and AFG_2_, the LB mean concentrations ranged from ND to 0.34 μg/kg and ND to 0.14 μg/kg, respectively. The UB mean concentration of AFG_1_ and AFG_2_ ranged from 0.17 to 0.82 μg/kg and from 0.17 to 0.29 μg/kg, respectively.

All four types of AFs were detected in rice, almonds, cashews, hazelnuts, peanuts, peanut butter, and spices, with total AFs ranging from 0.04 to 4.8 μg/kg and from 1.2 to 5.6 μg/kg for LB and UB concentrations, respectively. As expected, AFB_1_ was the most frequent AF type found in all food groups except for the spices where the LB and UB for AFG_2_ were found to be 1.0 and 1.6 μg/kg, respectively. The LB and UB mean concentrations of AFB_1_ in rice were found at 0.15 and 0.36 μg/kg. The highest mean LB and UB contamination of AFB_1_ was found in peanut butter samples at 3.8 and 3.9 μg/kg, respectively. The percentage of positive peanut butter samples contaminated with AFs was 82.4% (75 out of 91). Of the positive samples, six were domestic, 55 were imported, and the rest were of unknown origin. This was followed by sunflower seeds and peanuts, for which the percentages of positive samples were 34.8% and 26.2%, respectively. Interestingly, the AFs were ND in cakes, dessert mixes, groats, oats, pasta, popcorn, wheat, wheat flour, walnuts, cowpeas, lentils, and peas. Therefore, these food items were not included in the exposure assessment.

### 3.2 Exposure assessment

The results of the exposure assessment of AFs in the selected food groups and their contribution to the total dietary intake are presented in Table 4. Owing to the high proportion of left‐censored data in the present study, the exposure estimates were likely to be either underestimated or overestimated in the LB and UB scenarios, respectively. The total LB and UB dietary exposures to total AFs for the general population in the KSA through the consumption of the selected food items involved in this study were estimated at 0.29 and 1.84 ng/kg bw/day, respectively. Since AFB_1_ is proven to be the most toxic AF type, the EDIs of AFB_1_ based on LB and UB scenarios were calculated to be 0.21 and 0.55 ng/kg bw/day, respectively.

**TABLE 4:** The mean estimated daily intake of all four types of aflatoxins and total aflatoxins and the percentage of contribution to AFB_1_ specified by food group.

AF type	Food group					Total dietary intake of AFs (ng/kg bw/day)
	Mean EDI of AFs (ng/kg bw/day)	Cereals and grains	Nuts and seeds	Pulses, legumes, beans, and their products	Spices	
AFB_1_	LB	0.18	0.02	0.01	0.00	0.21
	UB	0.43	0.05	0.06	0.02	0.55
AFB_2_	LB	0.02	0.00	0.00	0.00	0.03
	UB	0.08	0.01	0.01	0.00	0.10
AFG_1_	LB	0.01	0.00	0.00	0.00	0.02
	UB	0.75	0.02	0.09	0.03	0.90
AFG_2_	LB	0.01	0.00	0.00	0.02	0.03
	UB	0.22	0.01	0.03	0.03	0.28
Total AFs	LB	0.22	0.02	0.02	0.02	0.29
	UB	1.48	0.09	0.19	0.09	1.84
Food group contribution to EDI to AFB_1_ (%)		84.73	8.26	6.18	0.83	100

Abbreviation: EDI, estimated daily intake; LB, lower bound; UB, upper bound.

The contribution of each food item to the mean EDI of AFB_1_ is shown in Table S2. This was calculated using the LB approach to minimize the impact of the LOD and LOQ values. Overall, cereal and grain products contributed the most to the mean chronic dietary exposure to AFB_1_ with a mean EDI of 0.178 ng/kg bw/day, accounting for 84.7% of the total LB mean exposure. Rice was the main contributor to the dietary intake of AFB_1_ in the cereal and grain groups, with a percentage of 83.3% and mean EDI of 0.175 ng/kg bw/day. This was followed by peanut butter, where the mean EDI of AFB_1_ was estimated at 0.01 ng/kg bw/day, accounting for 4.9% of the mean overall EDI of AFB_1_.

Moreover, the highest LB and UB mean EDI of AFG_1_ were estimated at 0.012 and 0.71 ng/kg bw/day through the consumption of rice. Interestingly, the highest LB mean EDI of AFG_2_ was 0.021 ng/kg bw/day in spices, whereas the highest UB mean EDI of AFG_2_ was estimated at 0.21 ng/kg bw/day from rice. To sum up, the total LB and UB mean EDI of AFG_1_ was 0.016 and 0.89 ng/kg bw/day, respectively. The mean overall EDI of AFG_2_ was 0.035 and 0.28 ng/kg bw/day in the LB and UB, respectively.

### 3.3 Risk characterization

The results of AFB_1_ risk characterization are presented in Table 5. The calculated MoE values were 1902.4 and 722.1 for the LB and UB mean EDI of AFB_1_, respectively. The estimated cancer risk values following the LB and UB mean EDI of AFB_1_ were 0.002 and 0.008 cancer cases/year/100,000 persons, respectively.

**TABLE 5:** Risk characterization of AFB_1_ based on margin of exposure and cancer risk approaches for the adult population in the Kingdom of Saudi Arabia.

EDI (ng/kg bw/day)		MoE		HCC risk estimates (cancer cases/year/100,000 persons)	
LB	UB	LB	UB	LB	UB
0.21	0.55	1902.38	722.08	0.002	0.008

Abbreviation: EDI, estimated daily intake; HCC, hepatocellular carcinoma; LB, lower bound; MoE, margin of exposure; UB, upper bound.

## 4 DISCUSSION

### 4.1 Occurrence of AFs

The AFB_1_, AFB_2_, AFG_1_, and AFG_2_ occurrence levels were determined in 2388 food samples. The levels of total AFs in the positive samples were within the limits determined by the Gulf Cooperation Council Standardization Organization (GCC Standardization Organization) except for two rice samples and five pistachio samples, three of which were imported, and the others were of unknown origin. The results showed a difference in AFB_1_ mean concentrations between peanuts and their product (peanut butter) where the levels found at 0.31 and 3.7 μg/kg, respectively, indicated that processing methods and storage conditions are very critical points to be monitored and controlled by food producing companies. Remarkably, AFB_1_ mean concentrations in peanut butter samples collected from Riyadh, Jeddah, and Dammam were detected at the same level of 3.7 μg/kg. This could be because peanut butter is considered a processed food that is distributed throughout the KSA under the same conditions, unlike rice where the AFB_1_ mean concentrations in samples collected from Jeddah and Dammam were considerably higher than those collected from Riyadh (0.27 and 0.07 μg/kg, respectively). This might be because humid climates in the eastern and western regions provide an optimal environment for fungal growth in raw materials, such as rice.

This study showed that nuts and seeds are the major food groups contaminated with AFs in the KSA. Our findings are in agreement with those of a recent study conducted in the KSA by Serdar et al. (Serdar et al., 2020), which measured AF levels in different food groups. However, their results were considerably higher than those obtained in our study, with mean total AF concentration values of 282.28 μg/kg in walnut samples and 12.7 μg/kg in pistachio samples. This could be due to the fact that all samples in their study were taken from the western region, which is known for its high temperature and humid weather most seasons of the year. Furthermore, El Tawila et al. (El Tawila et al., 2020) found that 27.3% and 20% of nut samples (*n* = 99) were contaminated with AFB_1_, AFB_2_, AFG_1_, and AFG_2_, respectively, which was much higher than the percentages of positive samples in our study (14%). The variations in concentrations between the two studies could be due to the nut moisture content, soil properties, and poor storage conditions, resulting in fungal growth. Ashraf (Ashraf, 2012) studied the occurrence of AFs in dry nuts consumed in the KSA and found that AFB_1_ was detected in 92% of cashew nuts, six of them exceeding the ML of 5 μg/kg set by the EU, whereas a lower contamination level was found in macadamia samples (~85% of the samples). In contrast, Bokhari (Bokhari, 2001) determined the levels of AFs in various food groups, such as cereal grains, spices, nuts, beans, and oilseeds, in the KSA. Similarly, the highest AFB_1_ levels were detected in oilseeds and cereal grains with mean concentrations of 12.9 and 8.02 μg/kg, respectively; however, they found lower levels in the beans and nuts (mean concentrations of 1.99 and 2.73 μg/kg, respectively). Surprisingly, another recent study measured the levels of the four AFs and the total AFs in rice, with all samples below the LOQ values (Almutairi et al., 2021). They justified the absence of AFs in nearly 480 samples with the overuse of fungicides by farmers, which could have affected the growth of the fungus in rice samples (Kumar et al., 2013).

On an international level, studies have measured the occurrence of AFs in food commodities (Cano‐Sancho et al., 2013; Ding et al., 2012; Hassan et al., 2022; Kang'ethe et al., 2017; Sugita‐Konishi et al., 2010; Thuvander et al., 2001; Zinedine et al., 2007). The total AF mean concentrations observed in the present study were much lower than the total AF concentrations reported in Spain, with a mean level of 2.7, 8.9, 0.5, and 0.9 μg/kg in peanuts, pistachios, breakfast cereals, and corn, respectively (Cano‐Sancho et al., 2013). In Sweden, the levels of total AF contamination were extremely high in pistachios and Brazilian nuts, with maximum values of 2200 and 2500 μg/kg, respectively (Thuvander et al., 2001). Additionally, very high levels of AFs have been reported in Morocco, where 50% of corn samples were contaminated with AFB_1_ at a mean concentration of 1930 μg/kg (Zinedine et al., 2007). In the same study, all red paprika samples (100%) were contaminated with AFB_1_ at a mean concentration of 2.88 μg/kg. In Lebanon, AFB_1_ was detected in all 105 collected white, parboiled, and brown rice samples with a mean AFB_1_ concentration of 0.5 μg/kg and a range of 0.06–2.08 μg/kg (Hassan et al., 2022), while the present study found lower levels of AFB_1_ in rice, with mean concentrations at 0.15 and 0.36 μg/kg in the LB and UB, respectively, as stated earlier in the study's results.

### 4.2 Exposure assessment

Although the highest mean AFs concentrations were found in the nut and seed groups, cereals and grain products made the largest contribution to the mean EDI of AFs. Moreover, cereal and grain products accounted for more than 80% of the overall mean EDI of AFB_1_. This might be due to the high consumption of rice (82.3 g per day) as one of the main staple foods in the KSA. In Japan, the EDI of AFB_1_ ranges from 0.003 to 0.004 ng/kg bw/day, whereas the EDI of total AFs ranges from 0.003 to 0.014 ng/kg bw/day, considering chocolate and peanut butter as the main contributors to the EDI of total AFs. This study concludes that the current dietary intake of AF in Japan is low and has no considerable effect on human health (Sugita‐Konishi et al., 2010). Another study assessed the EDI of the Catalonian population for AFs through the consumption of peanuts, pistachios, dried figs, sweet corn, breakfast cereals, and corn snacks (Cano‐Sancho et al., 2013). Their results illustrated that peanuts and pistachios were the highest contributors to the total AF intake in adult males and females, with a mean EDI of total AFs of 0.135 and 0.078 ng/kg bw/day, respectively. Additionally, another study examined 300 samples of hazelnuts and dried figs to estimate the EDI of AFB_1_ and total AFs in the Turkish population. The study found that the EDI of AFB_1_ and total AFs ranged from 0.003 to 0.016 ng/kg bw/day and from 0.004 to 0.023 ng/kg bw/day, respectively, and the contribution of hazelnuts to the EDI of total AFs was higher than that of dried figs (Kabak, 2016). By comparing the estimated total AFs intake of the Saudi adult population in the current study with the ranges mentioned above, it can be concluded that the EDI of total AFs in the KSA is higher, regardless of the lower concentration values. Nevertheless, Andrade et al. (Andrade et al., 2013) estimated considerably higher EDI values for the Brazilian population, with estimates ranging from 6.8 to 27.6 ng/kg bw/day, indicating a potential health concern. According to a report conducted by Shephard (Shephard, 2008) that examined AFs in Africa, the intake of the Kenyan population from consuming 400 g per day of maize was estimated to be 133 ng/kg bw/day, whereas the intake from peanuts was reported at 99.2 ng/kg bw/day in Ghana (Williams et al., 2004). In a previous study conducted in China, in which 1854 samples were analyzed to assess the probabilistic risk of dietary intake of AFB_1_ contamination in different food groups, the results revealed that rice and rice products were the main contributors to the dietary intake of AFB_1_. The EDI of AFB_1_ in the adult group was estimated at 0.59 ng/kg bw/day (Zhang et al., 2020).

In Serbia, the mean overall EDI of AFB_1_ among male and female adults was in the range of 0.56–0.76 ng/kg bw/day and 0.52–0.72 ng/kg bw/day for the LB and UB scenarios, respectively (Udovicki et al., 2021). These values are higher than the results reported in the present study. Table S2 illustrates the results of the exposure assessment for the four types of AFs and total AFs, taking into account the available consumption data.

### 4.3 Risk characterization

AFB_1_ is the most toxic AF type and is most frequently found in food commodities, whereas the other three types are rarely found in the absence of AFB_1_ (EFSA Panel on Contaminants in the Food Chain (CONTAM) et al., 2020). To conduct risk characterization estimates, the MoE approach was adopted to evaluate the risks related to human health associated with AFB_1_. As identified by the EFSA in their report on the risk assessment of genotoxic and carcinogenic substances, a small MoE indicates a higher risk than a larger MoE, where values below 10,000 represent a human health concern (European Food Safety Authority (EFSA), 2005). In this study, the estimated MoE values were 1902.4 and 722.1 for the mean LB and UB EDI of AFB_1_, respectively. By comparing the MoE range in the present study with the range provided by Shephard (Shephard, 2008), it can be anticipated that foods consumed in the KSA are less contaminated than those consumed in Africa, where most MoE values are below 10. In addition, our MoE value is considerably higher than that reported in Brazil, where the MoE value was estimated at 25 for the total population, indicating a potential risk for consumers, although the variety of food items collected and analyzed in this study was wider than that reported by Andrade et al (Andrade et al., 2013). Moreover, the MoE values estimated in a study conducted in China ranged from 24.1 to 1273 based on the AFB_1_ exposure estimate from the consumption of peanuts (Ding et al., 2012). As reported by Kabak (Kabak, 2021), the calculated MoE values ranged from 995 to 860 for adult Turkish consumers, based on the mean EDI of AFB_1_, which was assessed in five food groups. The above‐mentioned MoE ranges, including the calculated values in the current study, were all far lower than 10,000, which could be indicators of health concerns in long‐term exposure. It should be emphasized that the number of food items investigated in this study was greater than that in previous studies, which resulted in higher exposure estimates and therefore, lower MoE values.

Epidemiological studies suggest a correlation between AFB_1_ exposure and an increased incidence of primary liver cancer in the presence of other risk factors such as HBV infection (Liu et al., 2012). The estimated cancer risk values in the present study varied greatly when compared to the estimations from the EFSA's report on risk assessment of AFs in foods, where the cancer risk ranged between 0.004 and 0.057 cancer cases/year/100,000 persons for adults following LB and UB mean EDI of AFB_1_ (EFSA Panel on Contaminants in the Food Chain (CONTAM) et al., 2020). Moreover, the potential cancer risk at the 90th percentile in Japan is even lower, with estimates ranging from 0.00004 to 0.00005 cancer cases/year/100,000 persons (Sugita‐Konishi et al., 2010). In contrast, a very high population risk was estimated for the Ghanaian population, where it reached 70.1 cancer cases/year/100,000 persons (Shephard, 2008). Similarly, based on the average dietary intake of different food commodities contaminated with AFs in Bangladesh, the associated risk of HCC was estimated to be 1311 cancer cases/year/100,000 people, indicating a high risk to the population (Saha Turna & Wu, 2019). In conclusion, the estimated cancer risks in the general population following exposure to AFB_1_ were consistent with the conclusions drawn from the MoEs.

There are some shortcomings in the present study that should be addressed in future research. First, the exposure assessment was performed for the adult population as a single group, without considering sex‐based differences in food consumption patterns, weight status, and dietary habits. It is also important to note that a national food consumption database should be considered to provide a more realistic risk assessment. However, owing to the lack of these data in the KSA, an international food database was used, which may have either underestimated or overestimated the EDI of AFs and, consequently, the risk characterization.

## 5 CONCLUSIONS

The highest percentage of positive samples in the current study was found in spices. The highest concentration of total AFs was observed in the nut and seed groups, which were found in peanut butter. Future studies should use consumption data that are more accurate. This study used the GEMS/Food cluster diets database, which provides consumption data based on dietary patterns and food balance sheets provided by the FAO. We conclude that AFs contamination in the KSA is low; however, AFs are genotoxic carcinogens. Therefore, a monitoring program for these contaminants should be maintained and continuously improved. Controlling the commercial practices and storage conditions for specific products is essential to guarantee that human exposure levels are kept as low as possible, and household awareness should be raised regarding the handling of food that may be contaminated with AFs during storage.

## AUTHOR CONTRIBUTIONS

**Jumanah Alamir:** Data curation (equal); formal analysis (equal); investigation (equal); methodology (equal); project administration (lead); visualization (lead); writing – original draft (lead). **Lama Almaiman:** Data curation (equal); formal analysis (equal); investigation (equal); methodology (equal); writing – original draft (equal). **Yasser Alrujib:** Formal analysis (equal); methodology (equal); validation (equal); visualization (equal); writing – original draft (equal). **Rayan Alhamidi:** Data curation (equal); formal analysis (equal); methodology (equal); visualization (equal); writing – original draft (supporting). **Bandar Al‐owais:** Data curation (equal). **Saqer Alhussain:** Data curation (supporting). **Abdullah Aldakheelallah:** Writing – original draft (supporting); writing – review and editing (equal). **Majid AlKhalaf:** Writing – review and editing (equal). **Mohammed Bin Eid:** Conceptualization (lead); methodology (equal); project administration (equal); supervision (lead); writing – review and editing (equal).

## FUNDING INFORMATION

This research received no external funding.

## CONFLICT OF INTEREST STATEMENT

All the authors declare no conflict of interest, financial or otherwise.

## DISCLAIMER

The views expressed in this paper are those of the author(s) and do not necessarily reflect those of the SFDA or its stakeholders. Guaranteeing the accuracy and validity of the data is the sole responsibility of the research team. The authors are not aware of any affiliation, membership, funding, or financial holding that might be perceived as affecting the objectivity of this study.

## Supporting information


Tables S1–S2

## Data Availability

The data that support the findings of this study are available on request from the corresponding author. The data are not publicly available due to privacy or ethical restrictions.
